# The prospective association of adolescent loneliness and low resilience with anxiety and depression in young adulthood: The HUNT study

**DOI:** 10.1007/s00127-025-02888-2

**Published:** 2025-04-07

**Authors:** Nayan Parlikar, Linn Beate Strand, Kirsti Kvaløy, Geir Arild Espnes, Unni Karin Moksnes

**Affiliations:** 1 Department of Public Health and Nursing, Faculty of Medicine and Health Sciences, Norwegian University of Science and Technology, Trondheim, Norway; 2 HUNT Research Centre, Department of Public Health and Nursing, Norwegian University of Science and Technology, Levanger, Norway; 3 Levanger Hospital, Nord-Trøndelag Hospital Trust, Levanger, Norway

**Keywords:** Adolescence, Loneliness, Resilience, Interaction analyses, Prospective study, Mental health, Anxiety, Depression, HUNT Study

## Abstract

**Background:**

Loneliness is a recognized risk factor for anxiety and depression, yet research on its interaction with low resilience remains sparse, particularly across the adolescent-to-adult transition. This study investigates how adolescent loneliness, both independently and in interaction with low resilience, influences anxiety and depression in young adulthood.

**Methods:**

This study utilized longitudinal data from The Trøndelag Health Study (HUNT) to track adolescents (13–19 years) from Young-HUNT3 (2006-08) through to HUNT4 (2017-19). Loneliness was assessed via a single-item measure, while resilience was estimated using the Resilience Scale for Adolescents. Anxiety and depression outcomes at the 11-year follow-up were measured using the Hospital Anxiety and Depression Scale. Multivariable logistic regression analyses were done to analyze the associations. Moreover, interaction effects were evaluated using relative excess risk due to interaction (RERI).

**Results:**

Loneliness during adolescence independently predicted anxiety and depression in young adulthood. Adolescents experiencing both loneliness and low resilience showed notably higher risks compared to other groups (highly resilient adolescents without loneliness [reference], highly resilient adolescents with loneliness, and adolescents with low resilience and low loneliness). The combined effect of loneliness and low resilience exhibited a synergistic interaction on the additive scale, although it was not statistically significant (RERI 0.13, 95% CI -2.39-2.65).

**Conclusion:**

Adolescent loneliness and low resilience independently predict anxiety and depression in young adulthood. The interaction between loneliness and low resilience further heightens these risks. This underscores the importance of early interventions that focus on resilience-building during adolescence and reducing the impacts of loneliness on mental health.

**Supplementary Information:**

The online version contains supplementary material available at 10.1007/s00127-025-02888-2.

## Introduction

Mental health is fundamental to overall health, societal prosperity and global development [1, 2]. Anxiety and depression are the most debilitating mental health conditions worldwide, with over 300 million people affected by depression and around 45.82 million cases of anxiety disorders reported globally in 2019 [3–6]. These issues have been rising in Europe, particularly among adolescents and young adults, with around 13.6 million affected individuals in 2019, highlighting the need for global attention [7, 8]. The burden of mental illness in young adulthood often stems from factors experienced during adolescence, with loneliness being a significant, yet understudied factor [9, 10]. Through a deeper exploration of these dynamics, this study aims to illuminate the pathways linking adolescent loneliness to anxiety and depression in young adulthood.

Loneliness, characterized by a perceived lack of social connection compared to desired levels [11], regardless of social isolation status, can negatively affect individuals` life prospects. Research shows that loneliness represents a risk factor for depression [12, 13] and is also associated with anxiety [12, 14]. Research shows that loneliness can lead to a lack of social support and meaningful connections, further exacerbating feelings of distress and contributing to the onset of anxiety and depression [15]. While previous research has established the link between loneliness and mental health problems [16–18], there remains a gap in understanding the long-term effects of loneliness on mental health into adulthood [19]. Adolescent loneliness has gained public health attention recently due to its profound implications for individual and societal health [20, 21]. Adolescence is a phase of major biological, psychological, social, and cognitive transitions, making adolescents more vulnerable to loneliness than those in later life stages [22, 23], given their strive for independence from their families and the need to seek social and emotional connections with their peers [23]. A failure to form such intimate relationships during this developmental stage can heighten the risk of experiencing loneliness [24, 25]. The association between adolescent loneliness, anxiety and depression has been studied predominantly in cross-sectional studies [12, 26] and retrospectively [27, 28], but prospective studies with follow-up from childhood and adolescence to young adulthood are insufficient [29, 30]. Moreover, these limited prospective studies available have short follow-up periods, limiting the ability to capture long-term effects [18].

The impact of loneliness varies based on individual vulnerabilities, protective factors, and resources [31]. Resilience, a key protective factor, promotes healthy development by helping individuals cope with life’s challenges. Resilience refers to the process wherein an individual copes well to navigate life’s challenges by leveraging protective resources within themselves or their environment [32–35]. Resilience is a dynamic process, involving the ability to adapt and recover from adversity. It varies over time and situations, emphasizing the need for ongoing personal growth and support to cope with challenges [36]. Research shows resilience is negatively correlated with anxiety and depression,, suggesting it may protect against these conditions by buffering risk factors [37–43]. Low resilience, however, makes coping with stress harder, increasing vulnerability to mental health issues [40]. Despite extensive studies on childhood adversities, there is a gap in research on low resilience in adolescent loneliness and its impact on mental health. Understanding this relationship is key for effective interventions and promoting long-term well-being, especially in children and adolescents [44–46]. Therefore, we carried out a prospective cohort study of adolescents using The Trøndelag Health Study (HUNT) to (i) investigate the association of adolescent loneliness and low resilience with anxiety and depression in young adulthood and (ii) assess the joint association of adolescent loneliness and low resilience with anxiety and depression in young adulthood.

## Materials and methods

### Study population

The study used data from The Trøndelag Health Study (HUNT), a large population-based cohort in Trøndelag County, Norway [47–49]. The HUNT Study consists of four health surveys carried out at 11-year intervals, with Young-HUNT targeting adolescents (13–19 years) and HUNT for adults (20 + years) from the Trøndelag County in Norway. The Young-HUNT Study comprises three different waves: Young-HUNT1 (1995-97), Young-HUNT3 (2006-08), and Young-HUNT4 (2017-19) [47]. Adolescents from Young-HUNT3 (YH3) were included, with data collected via questionnaires, exams, and interviews. Adolescents not attending the school on the day of assessment received the questionnaire by mail [47]. The HUNT4 (2017-19) Survey took place 11 years later. The participants received an invitation letter and were asked to complete the electronic questionnaire and undertake physical tests at a local field station [49]. Of the 8199 adolescents who were included in YH3, 2293 (29%) attended HUNT4. Participation in the HUNT Study was voluntary, and all participants signed a written consent form. Written consent from parents or a guardian was required for participants under the age of 16 years. The Regional Committee for Medical and Health Research Ethics (Ref number: 286734) approved the present study.

### Variables

#### Exposures variables at baseline; Young-HUNT3 (2006-08)

##### Loneliness

Loneliness was measured with the question, “Are you lonely?” on a five-point scale from " [1] Very rarely or never” to “ [5] Very often.“. The loneliness variable was grouped in two ways. Firstly, for the purpose of the multivariable logistic regression, categories 1 and 2 were grouped together to make a single category, “No,” with participants who reported low levels of loneliness. The other categories were kept unchanged. Secondly, the loneliness variable was dichotomized into the category Rarely lonely (‘Very rarely or never’ + ‘Rarely’ + ‘Sometimes lonely’) and the category Very lonely (‘Often’ + ‘Very often’) for the purposes of the interaction analyses. The dichotomization of the variable of loneliness has been previously utilized in studies focusing on adolescent populations [50–52]. The ‘Very lonely’ category was presumed to capture the most severe and high-risk cases of loneliness, and the ‘Rarely lonely’ category captured less severe and temporary forms or a complete absence of loneliness.

##### Resilience

Resilience factors were measured with 8 items from the Resilience Scale for Adolescents (READ) [53] in YH3, addressing social competence and family cohesion. Social competence items included items: I easily make others feel comfortable around me; I easily find new friends; I am good at talking to new people; I always find something fun to talk about and family cohesion: In my family we share views of what is important in life; I feel comfortable with my family; My family views the future as positive, even when sad things happen; In my family, we support each other. Responses ranged from [1]‘I totally disagree’ to [5]‘I totally agree’, with higher scores indicating higher resilience. The READ provides an exciting possibility to assess several different resilience factors with relatively few items, and can thus be used as a valuable measurement tool in resilience and risk factor research [54]. Furthermore, several studies support the use of the scale during all of adolescence, from 14 to 15 years of age up to 18 to 20 years of age. The scale may also be relevant in more applied settings, where it could be used to identify strengths and weaknesses in life areas that may enhance resilient outcomes in adolescents exposed to risk [54–56].

#### Demographics and baseline mental health

Covariables included age at baseline (13–15 and 15.1–19 years), gender, socioeconomic status (SES) at baseline, quality of relationship with family members, school satisfaction, and baseline self-esteem. Hopkins Symptom Checklist-25 (HSCL-25) was used as a control variable for the sensitivity analyses.

SES was assessed with the question: “How well off do you think your family is compared to most others?” Responses were: [1] ‘About the same as most others,’ [2] ‘Better,’ and [3] ‘Worse.’ The quality of family relationships was measured by: “How good is the relationship with your immediate family?” with responses from [1] ‘Very good’ to [4] ‘Bad.‘. School satisfaction was measured with nine questions on topics like concentration, arguing with teachers, school enjoyment, skipping school, understanding lessons, boredom, teasing, exclusion, and exam satisfaction, with responses from [1] ‘Never’ to [4] ‘Very often.‘. Self-esteem was addressed by the Rosenberg Self-esteem Scale [57]. This scale covered the questions: I have a positive attitude towards myself; I certainly feel useless at times; I feel I do not have much to be proud of; I feel that I’m a person of worth, at least on an equal plane with others. Responses ranged from [1] ‘I totally agree’ to [4] ‘I totally disagree’. The negatively phrased items on the school satisfaction and self-esteem scale were reverse-coded. Then, the scores were averaged and dichotomized. Scores from 1 to 2.99 were considered as low school satisfaction and self-esteem, while scores above 2.99 were considered as high. Age groups are displayed as 13–15 years and 16–19 years in tables. HSCL-5, a 5-item version of the HSCL-25, an extensively used self-report measure of anxiety and depression symptoms among adolescents, was used for sensitivity analyses. Compared with the HSCL-25, the short-form 5-item HSCL has acceptable validity [58]. The adolescents were asked if they had experienced each of the following during the last 14 days: Been constantly afraid and anxious, Felt tense or uneasy, Felt hopelessness about the future, Felt dejected or sad, Worried too much about various things. Each item was answered on a four-point scale: [1] ‘Not at all’ to [4] ‘Very much’ with the cut-off score ≥ 2.

#### Outcome variables; HUNT4 (2017-19)

##### Anxiety and depression

Symptoms of anxiety and depression were measured with the Hospital Anxiety and Depression Scale (HADS), which consists of 14 questions of which seven measure anxiety symptoms and seven measure depression symptoms, during the past week. Each question is answered on a scale of 0–3, giving one total score for anxiety (HADS-A, range 0–21) and one total score for depression (HADS-D, range 0–21). A total score from 0 to 7 indicates normal state, 8 to 10 indicates borderline anxiety or depression state and 11–21 indicates symptomatology of anxiety or depression. In the analysis stratified by resilience level, we combined the last two categories (score 8–21) to define the presence of symptoms (yes, no), i.e., a cut-off score ≥ 8. A total HADS score (HADS-T) consists of the sum of the HADS-A and the HADS-D scores, and the range is from 0 to 42 points. The cut-off indicating HADS-T symptomatology in this study was set at 19 points as recommended in the literature [59, 60]. Additional details of HADS are described elsewhere [61, 62]. The HADS was used to describe the presence of symptoms and is not a psychiatric diagnosis.

### Statistical analyses

In the study sample of 2293 participants, we evaluated loneliness at baseline and the prospective outcome of anxiety and depression in relation to other baseline characteristics. We computed the mean baseline resilience scores by aggregating responses to the set of eight questionnaire items. We then divided participants into high and low-resilience groups, delineating low resilience at the threshold of ≤ 25th percentile (cut off: 3.625). The dichotomization of resilience items has been done before in previous studies among adolescents [63–65]. Categorical variables describing the study samples were reported as frequencies and percentages. To study the association of loneliness and resilience with anxiety and depression, we estimated odds ratios (OR) with 95% confidence intervals (CIs) using multivariable logistic regression. The analyses were conducted separately for each of the three outcomes, i.e., anxiety (HADS-A), depression (HADS-D), and combined measures of anxiety and depression (HADS-T) with the dichotomized exposures of loneliness and resilience. We reviewed the literature and created Directed Acyclic Graphs (DAGs) to select covariates that could be associated with loneliness (exposure) and anxiety and depression (outcome) in the regression analyses.

In Model I, we adjusted for age and gender. Model II was adjusted for age, gender, SES, quality of relationship with family members, school satisfaction, and self-esteem as potential confounding factors. In Model III, we additionally adjusted for resilience and loneliness as continuous variables. To assess the joint association of adolescent loneliness and low resilience with anxiety and depression in young adulthood, we generated four subgroups, i.e. participants with high resilience without loneliness (reference group), participants with high resilience and with loneliness (ORA), participants with low resilience without loneliness (ORB) and participants with low resilience with loneliness (ORAB). Logistic regression analyses were conducted to examine the relationship between adolescent resilience and anxiety and depression in young adulthood. The joint associations of loneliness and low resilience with anxiety and depression were estimated by using the relative excess risk due to interaction (RERI) with 95% CI. The RERI was calculated using an additive model: RERI = ORAB– ORA– ORB + 1 [66]. The additive model is used to test the interaction between two or more risk factors that together assert their influence on disease risk [67–69]. In brief, RERI > 0 and the lower limit of 95% CI > 0 suggest a synergistic effect of loneliness and low resilience on anxiety and depression [69]. Moreover, in addition to interaction on the additive scale, we assessed interaction on the multiplicative scale as it is always best to present both the additive and multiplicative measures of interaction [69].

#### Sensitivity analyses

We performed sensitivity analyses by classifying participants in two ways. First, those scoring ≥ 8 on HADS-A or HADS-D were considered to exhibit symptoms of both anxiety and depression (*N* = 1443) [70–73]. Second, participants who had utilized healthcare—reported psychiatrist visits, polyclinic treatments in the past year, or intake of antidepressants and anxiolytics in the past month—along with those scoring ≥ 8 on either HADS set, were also considered to exhibit symptoms (*N* = 806). To address reverse causality, we included only those adolescents who scored < 2 on the HSCL-5 in YH3 (free from anxiety and depression at baseline) in another sensitivity analysis. This group included 1826 adolescents (22.27%). We repeated all statistical analyses in this baseline symptom-free sample.

All analyses were done using R.

## Results

Among the 2293 participants, the prevalence of loneliness was 8.2% (*N* = 189). During the 11-year follow-up, anxiety prevalence was 16.6% (*N* = 380) and depression prevalence was 6.1% (*N* = 141) (Table 1). Loneliness was more prevalent among girls, 16–19-year-olds, the mentally distressed (HSCL ≥ 2), those with low resilience, poor family relationships, low self-esteem, low school satisfaction, and low SES. Anxiety and depression at follow-up were higher in women, 23–25-year-olds, the mentally distressed, those with low resilience, poor family relationships, low self-esteem, low school satisfaction, and low SES.

**Table 1:** Baseline (2006–2008) characteristics of the study sample stratified on loneliness and symptoms of anxiety and depression in the follow-up (2017–2019)

Variables measured at baseline	Total	Young-HUNT3 2006–2008 (Baseline)		HUNT4 2017–2019 (Follow-up)			
		Loneliness		Anxiety symptoms		Depression symptoms	
		No.	%	No.	%	No.	%
Study cohort	2293	189	8.2	380	16.6	141	6.1
Missing		159	6.9	844	36.8	844	36.8
**Gender**							
*Girls*	1320	135	10.7	278	30	91	9.8
*Boys*	973	54	6.2	102	19.7	50	9.7
**Age**							
*13–15 years*	857	55	7.1				
*16–19 years*	1436	134	10				
*23–25 years*	501			102	31.5	43	13.3
*26–29 years*	1792			278	24.7	98	8.7
**Adolescent mental health**							
*HSCL ≥ 2*	434	135	33.5	132	46.8	52	18.5
*HSCL < 2*	1826	54	3.1	243	21.3	87	7.6
**Resilience**							
*Low*	626	141	23.8	143	35	69	16.8
*High*	1607	47	3.1	226	22.7	68	6.8
**Relationship with family**							
*Bad*	50	17	34.7	15	41.7	13	37.1
*Good*	2102	172	8.3	340	25.6	118	8.9
**Self-esteem**							
*Low*	832	160	20.4	208	37.5	86	15.5
*High*	1419	29	2.2	166	19.2	53	6.1
**School satisfaction**							
*Not satisfied*	448	100	22.7	100	37.6	49	18.4
*Satisfied*	1687	85	5.1	252	23.2	77	7.1
**Socio-economic status**							
*Low*	179	46	26.1	44	37.6	20	16.8
*Middle-class*	334	22	6.7	66	30.1	20	9.1
*High*	1626	118	7.3	244	23.9	88	8.6

Adolescents within the loneliness variable category “Quite a lot” and “Very much” Hospital Anxiety and Depression scale ≥ 8

### Associations of adolescent loneliness and anxiety and depression in young adulthood

We found an association between adolescent loneliness and anxiety and depression in young adulthood (Table 2). Participants with “Often” and “Very often” loneliness at baseline had an OR of 3.53 (95% CI 2.13–5.86) and 3.71 (95% CI 2.13–6.47) respectively for anxiety (HADS-A), after adjusting for age and gender. After adjustment for additional confounders (Model II), the OR was reduced to 2.52 (95% CI 1.47–4.34) and 2.42 (95% CI 1.32–4.45). Further adjustment for resilience (Model III) resulted in lower OR. Similar associations were noted for both depression (HADS-D) and the total measures of anxiety and depression (HADS-T).

**Table 2:** The association of loneliness in adolescence with anxiety and depression in young adulthood

Loneliness	Total	*N* (%)	Model I OR (95% CI)	Model II OR (95% CI)	Model III OR (95% CI)
**Anxiety (HADS-A)**					
*No*	906	182 (20)	Ref	Ref	Ref
*Sometimes*	325	111 (34.2)	2.0 (1.5–2.65)	1.54 (1.14–2.1)	1.51 (1.11–2.06)
*Often*	68	32 (47)	3.53 (2.13–5.86)	2.52 (1.47–4.34)	2.38 (1.37–4.13)
*Very often*	56	28 (50)	3.71 (2.13–6.47)	2.42 (1.32–4.45)	2.28 (1.23–4.23)
**Depression (HADS-D)**					
*No*	905	63 (7)	Ref	Ref	Ref
*Sometimes*	325	41 (12.6)	1.99 (1.31–3.04)	1.34 (0.84–2.14)	1.26 (0.78–2.01)
*Often*	68	15 (22)	4.18 (2.21–7.92)	2.45 (1.2–4.99)	2.08 (1.01–4.29)
*Very often*	57	12 (21)	3.61 (1.8–7.25)	1.45 (0.64–3.3)	1.25 (0.54–2.87)
**Anxiety and Depression (HADS T)**					
*No*	911	48 (5.3)	Ref	Ref	Ref
*Sometimes*	329	37 (11.2)	2.24 (1.42–3.53)	1.5 (0.9–2.49)	1.43 (0.86–2.38)
*Often*	68	11 (16.2)	3.71 (1.81–7.59)	2.39 (1.08–5.26)	2.08 (0.93–4.64)
*Very often*	57	12 (21.1)	4.44 (2.18–9.05)	2.25 (0.99–5.12)	1.98 (0.86–4.56)

Note CI, confidence interval; HADS-T, Total Hospital Anxiety and Depression scale; OR, odds ratio

HADS-A scale ≥ 8

HADS-D scale ≥ 8

HADS-T scale ≥ 19

Model I adjusted for age, gender

Model II adjusted for age, gender, relationship with family, SES, school satisfaction, and self-esteem at baseline. Model III additionally adjusted for resilience as a continuous variable

### Associations of adolescent resilience and anxiety and depression in young adulthood

We found an association between adolescents’ low resilience and anxiety and depression in young adulthood (Table 3). Participants with low resilience at baseline had an OR of 1.79 (95% CI 1.39–2.31) for anxiety (HADS-A) after adjusting for age and gender. After adjustment for confounders (Model II), the OR was reduced to 1.23 (95% CI 0.91–1.66). Additional adjustment for loneliness (Model III) reduced the OR even more. Similar associations were noted for depression (HADS-D) and for total measures of anxiety and depression (HADS-T).

**Table 3:** The association of resilience in adolescence with anxiety and depression in young adulthood

Resilience	Total	*N* (%)	Model I OR (95% CI)	Model II OR (95% CI)	Model III OR (95% CI)
**Anxiety (HADS-A)**					
*High*	997	226 (22.7)	Ref	Ref	Ref
*Low*	410	143 (35)	1.79 (1.39–2.31)	1.23 (0.91–1.66)	1.07 (0.78–1.47)
**Depression (HADS-D)**					
*High*	997	68 (6.8)	Ref	Ref	Ref
*Low*	410	69 (16.8)	2.85 (1.99–4.09)	1.73 (1.11–2.7)	1.57 (0.99–2.49)
**Anxiety and Depression (HADS-T)**					
*High*	1004	54 (5.4)	Ref	Ref	Ref
*Low*	415	59 (14.2)	2.91 (1.96–4.3)	1.66 (1.03–2.7)	1.48 (0.9–2.44)

Model I adjusted for age, gender

Model II adjusted for age, gender, relationship with family, SES, school satisfaction, and self-esteem at baseline. Model III additionally adjusted for loneliness

Low resilience delineated at the threshold of ≤ 25th percentile (3.625)

### Joint associations of adolescent loneliness and low resilience with anxiety and depression in young adulthood

**Fig. 1:**
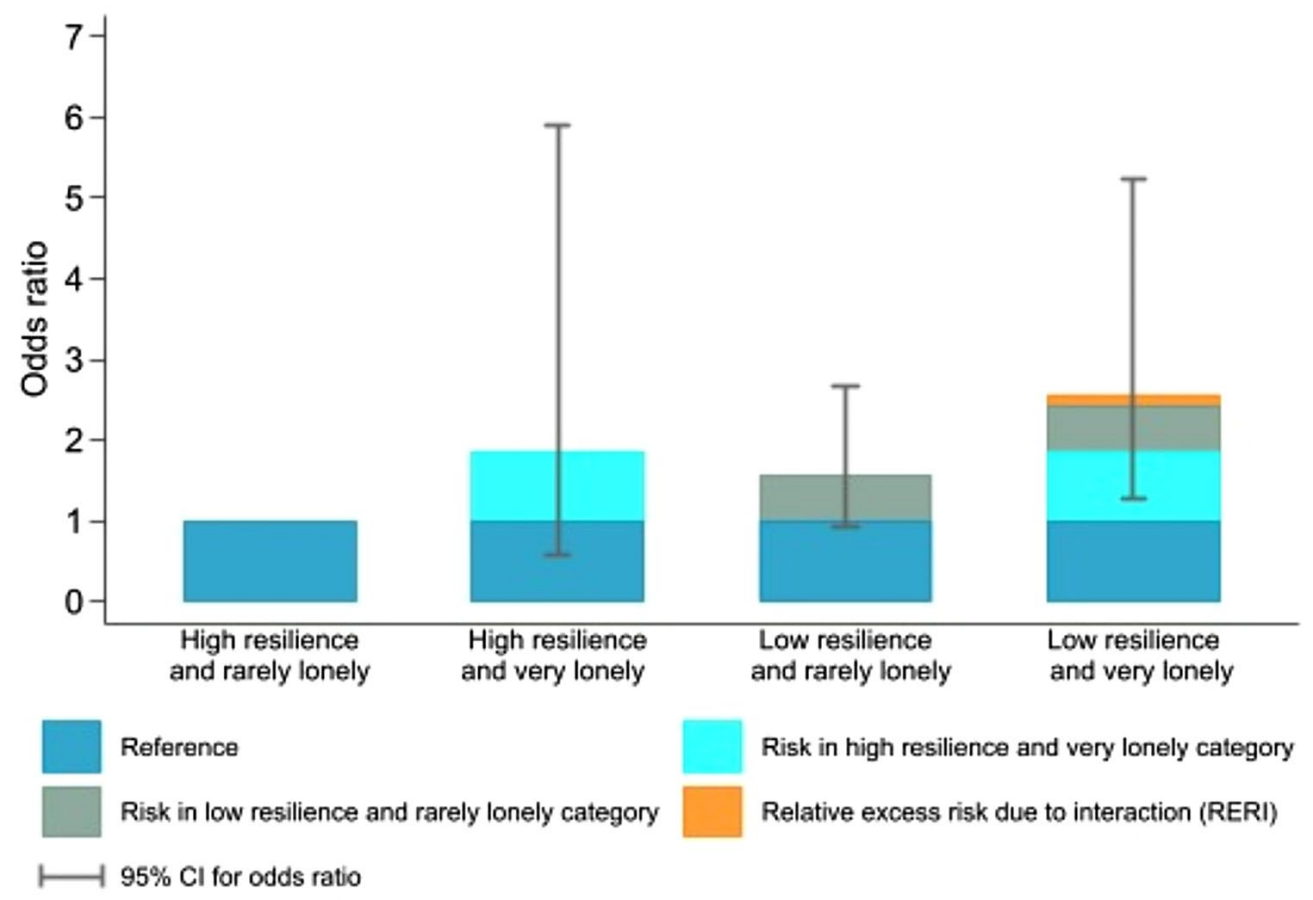
The risk of anxiety and depression in young adulthood associated with individual and joint exposures of adolescent loneliness and low resilience. Measurement of anxiety and depression levels in the study group, exclusively assessed using the Hospital Anxiety and Depression Scale Total (HADS-T). A HADS-T score of ≥ 19 indicates significant levels of anxiety and/or depression

Table 4 shows the joint association of adolescent loneliness and low resilience with anxiety and depression in young adulthood. For anxiety (HADS-A), lonely individuals with high resilience had an OR of 2.05 (95% CI: 0.96–4.35), low resilience without loneliness had an OR of 1.13 (95% CI: 0.81–1.58), and those with both had an OR of 2.11 (95% CI: 1.28–3.47). For depression (HADS-D), lonely individuals with high resilience had an OR of 2.36 (95% CI: 0.87–6.39), low resilience without loneliness had an OR of 1.81 (95% CI: 1.11–2.93), and those with both had an OR of 2.31 (95% CI: 1.17–4.55). For the total score (HADS-T), lonely individuals with high resilience had an OR of 1.87 (95% CI: 0.59–5.90), low resilience without loneliness had an OR of 1.57 (95% CI: 0.92–2.67), and those with both had an OR of 2.57 (95% CI: 1.26–5.23). We found a positive interaction on the additive scale for loneliness and resilience at baseline with the subsequent risk of anxiety and depression (HADS-T), with a RERI of 0.13 (95% CI -2.39-2.65), indicating the combined effect was greater than the sum of their individual effects, though not statistically significant. However, on the multiplicative scale, the interaction was negative for anxiety, depression, and the total score, meaning the combined effect was less than the product of their individual effects (Fig. 1).

**Table 4:** Adjusted odds ratios for adolescent loneliness and low resilience at baseline associated with anxiety and depression in young adulthood: additive and multiplicative interaction

Loneliness	High resilience			Low Resilience			RERI (95% CI)¹
	Anxiety symptoms (HADS-A)						
	Total	N	OR (95% CI)	Total	N	OR (95% CI)	
*Rarely lonely*	925	201	1.00	296	91	1.13 (0.81–1.58)**ORB**	-0.07 (-1.83-1.69)
*Very lonely*	32	14	2.05 (0.96–4.35)**ORA**	92	46	2.11 (1.28–3.47)**ORAB**	
**Depression symptoms (HADS-D)**							
*Rarely lonely*	925	58	1.00	295	45	1.81 (1.11–2.93)**ORB**	-0.87 (-3.49-1.76)
*Very lonely*	32	7	2.36 (0.87–6.39)**ORA**	93	20	2.31 (1.17–4.55)**ORAB**	
**Anxiety and Depression (HADS-T)**							
*Rarely lonely*	931	48	1.00	299	36	1.57 (0.92–2.67)**ORB**	0.13 (-2.39-2.65)
*Very lonely*	32	4	1.87 (0.59–5.90)**ORA**	93	19	2.57 (1.26–5.23)**ORAB**	

¹The relative excess risk due to interaction (RERI) between adolescent loneliness (A) and low resilience (B) was calculated using the formula: RERI = ORAB– ORA– ORB + 1

Adjusted for age, gender, relationship with family, SES, school satisfaction, and self-esteem at baseline

Measure of interaction on the multiplicative scale (HADS-A): 0.91 (95% CI. 0.37–2.23)

Measure of interaction on the multiplicative scale (HADS-D): 0.54 (95% CI 0.18–1.82)

Measure of interaction on the multiplicative scale (HADS-T): 0.88 (95% CI 0.26–3.62)

### Sensitivity analyses

The results of the sensitivity analyses and supplementary tables (S1-S7) are attached in separate files.

## Discussion

In this study, adolescents reporting loneliness and low resilience showed an increased risk of anxiety and depression symptoms in young adulthood after an 11-year follow-up. The combination of loneliness and low resilience was synergistic and further elevated this risk in young adulthood. These findings align with previous research linking adolescent loneliness to higher anxiety and depression risk across all ages. For instance, Stickley et al. [26] reported higher odds of headaches, anxiety, and depressive symptoms among lonely adolescents. Moreover, a multilevel meta-analysis by Maes et al. [74] found a positive association between loneliness and social anxiety symptoms across childhood and adolescence. Additionally, Beutel et al. [12] highlighted a strong association between loneliness and generalized anxiety and depression, especially among middle and late adulthood individuals. Our findings align with studies by Kehusmaa et al. [29] and Xerxa et al. [30], linking childhood and adolescent loneliness with anxiety and depression in young adulthood. Moreover, sensitivity analyses are further supported by studies on healthcare utilization, such as Beutel et al. [12], who found lonely individuals more likely to seek treatment for anxiety and depression. Similarly, Junker et al. [75] showed that loneliness, often accompanied by anxiety and depression, increased self-harm hospitalization risk among Norwegian adolescents. Moreover, Modin et al. found that low childhood social status, reflecting loneliness, predicted higher hospitalization risk for anxiety and/or depression in girls [76]. The pathway between loneliness and anxiety and depression involves psychological, biological, and social factors [77, 78]. Loneliness can lead to negative cognitive patterns, such as rumination and self-criticism, which are linked to anxiety and depression [14, 79]. Moreover, the chronic stress associated with loneliness dysregulates biological systems, including the hypothalamic-pituitary-adrenal (HPA) axis and the immune system, which are crucial in the onset and persistence of anxiety and depression [80–82]. Loneliness affects youth’s diurnal cortisol, a key stress hormone [83, 84], potentially causing chronic increases in cortisol in young adulthood, leading to higher risks of depression, anxiety, inflammation, and other adverse health outcomes [85–87]. Adolescents’ heightened exposure to loneliness can be explained by their strive for independence from their families and seeking social and emotional connections with their peers [23]. Failure to form such intimate relationships during this developmental stage can increase the risk of experiencing loneliness [24, 25]. Chronic loneliness during adolescence can hinder social skill development, leading to low self-esteem and vulnerability and thus exacerbating anxiety and depression [88]. Moreover, loneliness disrupts cognitive processes, increasing susceptibility to anxiety and depression [89]. Lonelier individuals may exhibit disrupted emotional regulation, leading to increased negative emotions and a higher propensity for risky health behaviors, which could impact future mental health outcomes [90]. Additionally, lonelier individuals experience poorer sleep quality in young adulthood, potentially worsening their long-term mental health [91].

This study also examines low resilience as an additional risk factor in the pathway between adolescent loneliness, anxiety, and depression in young adulthood. Several cross-sectional studies across different age groups have highlighted resilience’s protective role. For instance, Zhao et al. found resilience mediated the link between loneliness and depression in the elderly, while studies on working-age adults and Norwegian adolescents showed resilience moderated this relationship [92–94]. In contrast to the protective effect, the detrimental effect of low resilience was shown by Fangyan et al. [95]. They compared depression levels among young adults with high and low resilience, showing that higher loneliness correlated with increased depression among those with low resilience, likely due to stress hormones like cortisol, which can lead to long-term neurological and physiological issues [96]. Moreover, individuals with low psychological resilience seem to have compromised control of brain circuits involved in emotion and fear [97] and poor cortico-limbic inhibition, representing a dysregulated emotional response to stress that may result in elevated cortisol exposure [98]. Likewise, loneliness and social isolation during adolescence precipitate a state of vulnerability or social frailty [80], rendering individuals more susceptible to adverse outcomes. This vulnerability may arise from a lack of protective factors like familial, social, or communal support, which is crucial for managing life’s pressures. Consequently, loneliness and isolation can diminish resilience, leaving individuals more vulnerable to anxiety and depression [99]. Our study builds on prior research with a large prospective cohort and an 11-year follow-up, adjusting for various covariates. While we found that adolescent loneliness and low resilience were both associated with an increased risk of anxiety and depression in adulthood, the evidence for a synergistic effect was weak. The interaction analyses yielded a small RERI value (0.13) with wide confidence intervals, suggesting uncertainty in the estimate. While this may indicate a potential additive interaction, the results should be interpreted carefully. The mechanisms underlying these associations remain complex, potentially involving compromised stress-coping mechanisms in lonely individuals with low resilience, leading to worsening social interactions and prolonged loneliness. Further research is needed to confirm causal relationships and to explore additional factors that may influence these dynamics.

### Strengths and limitations

Our study’s strengths include a prospective design that establishes a clear temporal sequence, a large sample size at over an 11-year follow-up, and the ability to assess the interaction between adolescent loneliness and low resilience on young adult anxiety and depression. Additionally, the comprehensive dataset al.lowed control for various potential confounders. Resilience among adolescents was measured by the validated READ scale, covering relevant protective elements within resilience [54]. Similarly, anxiety and depression among young adults were measured by using the well-validated HADS questionnaire [61]. We conducted sensitivity analyses to ensure robustness, using an alternative definition of anxiety and depression that included those on antidepressant medication and those with recent healthcare visits, which increased precision and avoided misclassification. Additionally, we repeated all analyses after excluding adolescents who reported anxiety and depression at baseline.

This study has limitations. Self-reported measures may introduce biases such as social desirability and underreporting, while non-participation and absenteeism on data collection days may cause selection bias, potentially underestimating loneliness and its link to anxiety and depression. Despite an 11-year follow-up, more frequent assessments could better capture changes in loneliness and resilience. Self-reported loneliness is also prone to underreporting due to social stigma [100]. Loneliness is complex and ideally should be measured with a multi-item scale to account for variations in intensity, circumstances, and time with direct and indirect questions. Nevertheless, self-reported measures of loneliness have been shown to be equally reliable and valid as other forms of assessment [101]. Another issue is the categorization and applied dichotomization of loneliness and resilience. However, dichotomized outcomes may be necessary to communicate a comparison of risks [102]. Moreover, it is reassuring that this same dichotomization has been applied in earlier research among adolescents [50, 51, 103]. Moreover, despite the broad ranking and categorization of loneliness in this study, it fails to distinguish between adolescents who are rarely lonely and those who are never lonely. Another limitation is residual confounding despite controlling for relevant factors. Furthermore, it is uncertain if loneliness, low resilience, anxiety, and depression at baseline mutually influenced each other and thus increased the risk of anxiety and depression prospectively. Nevertheless, we excluded the adolescents who reported a score of ≥ 2 on the HSCL-5 at baseline in the sensitivity analyses to limit the possibility of reverse causation. In the current study, the HADS was used as a screening tool to capture symptoms of anxiety or depression and not a clinical diagnosis [104]. Although we strengthened the definition of anxiety and depression by adding medication use and hospital admission, we cannot rule out possible misclassification bias as we did not perform any objective tests. Thus, the limitations in identifying clinical anxiety or depression in adolescence and young adulthood, respectively, may limit the generalizability of our results.

We recognize the grade of attrition between the two surveys. However, this is a common issue in longitudinal studies [105, 106]. Several factors contributed to this: the long follow-up period (over 11 years), during which participants relocated or lost interest in follow-up studies, lower participation rates among young adults, as previously documented in HUNT research [47, 48], and relocation or death [107]. Moreover, the tendency of migration from rural Nord-Trøndelag to urban areas contributed to attrition [108, 109]. Mental health may have played a role, with those experiencing anxiety or depression being less likely to participate [110]. HUNT’s voluntary nature further challenges retention, and a lower response rate to the second questionnaire (Q2) in HUNT4 led to missing data on anxiety and depression (HADS) [49]. Despite this, strong baseline participation, consistent response patterns, and linkage to national registries support the study’s validity. Due to substantial missing data, we used complete case analysis rather than multiple imputation (MI) or weighting. MI assumes data are missing at random (MAR), but missingness in loneliness and anxiety/depression was likely due to participants experiencing these conditions, making MI unreliable [111]. Weighting does not fully remove bias when missingness relates to the outcome [111]. Non-responders were more likely to be older, male, or from vocational schools, suggesting socioeconomic differences [47], but these were already adjusted for in our models. Lastly, we adjusted for major confounders to account for potential bias due to missing data. But some level of bias might still be present. 

## Conclusion

In conclusion, our extensive cohort study indicates that adolescent loneliness and low resilience independently contribute to anxiety and depression in young adulthood. Low resilience interacting with loneliness can heighten this risk, reflecting the interconnectedness of the biological stress response. Our study underscores the importance of addressing loneliness and building resilience in adolescents to mitigate future anxiety and depression. Further research is needed to confirm the synergy between these risk factors and understand the mechanisms, potentially shifting resilience research toward a more holistic approach.

## Electronic supplementary material

Below is the link to the electronic supplementary material.

Supplementary Material 1

## Data Availability

The HUNT databank provided the data used in this study, but access is restricted. Data can be accessed by approaching the author and permission from HUNT, The Regional Ethical Committee, and the Norwegian Data Protection Authority. All the subjects of this study are stored in the HUNT data using a personal identification number as an I.D. The HUNT Research Centre stores and uses these data with authorization from the Norwegian Data Inspectorate without breaking participant privacy. The researcher will always receive an anonymous dataset after approval from the Regional Ethical Committee and HUNT Research Centre. For more information about HUNT data see https://www.ntnu.edu/hunt/data.

## References

[1] Organization WH, Mental (2022) health. https://www.who.int/news-room/fact-sheets/detail/mental-health-strengthening-our-response

[2] Nations U (2016) Health-United Nations Sustainable Development

[3] Vos T, Lim SS, Abbafati C et al (2020) Global burden of 369 diseases and injuries in 204 countries and territories, 1990–2019: a systematic analysis for the global burden of disease study 2019. Lancet 396(10258):1204–1222 33069326 10.1016/S0140-6736(20)30925-9 PMC7567026

[4] Collaborators GMD (2022) Global, regional, and National burden of 12 mental disorders in 204 countries and territories, 1990–2019: a systematic analysis for the global burden of disease study 2019. Lancet Psychiatry 9(2):137–150 35026139 10.1016/S2215-0366(21)00395-3 PMC8776563

[5] Liu Q, He H, Yang J, Feng X, Zhao F, Lyu J (2020) Changes in the global burden of depression from 1990 to 2017: findings from the global burden of disease study. J Psychiatr Res 126:134–140 31439359 10.1016/j.jpsychires.2019.08.002

[6] Yang X, Fang Y, Chen H et al (2021) Global, regional and National burden of anxiety disorders from 1990 to 2019: results from the global burden of disease study 2019. Epidemiol Psychiatric Sci 30:e36 10.1017/S2045796021000275 PMC8157816 33955350

[7] Castelpietra G, Knudsen AKS, Agardh EE et al (2022) The burden of mental disorders, substance use disorders and self-harm among young people in Europe, 1990–2019: findings from the global burden of disease study 2019, vol 16. The Lancet Regional Health–Europe 10.1016/j.lanepe.2022.100341 PMC8980870 35392452

[8] Krokstad S, Weiss DA, Krokstad MA et al (2022) Divergent decennial trends in mental health according to age reveal poorer mental health for young people: repeated cross-sectional population-based surveys from the HUNT study, Norway. BMJ Open 12(5):e057654 35584877 10.1136/bmjopen-2021-057654 PMC9119156

[9] Solmi M, Radua J, Olivola M et al (2022) Age at onset of mental disorders worldwide: large-scale meta-analysis of 192 epidemiological studies. Mol Psychiatry 27(1):281–295. 10.1038/s41380-021-01161-7 34079068 10.1038/s41380-021-01161-7 PMC8960395

[10] Beutel ME, Klein EM, Brähler E et al (2017) Loneliness in the general population: prevalence, determinants and relations to mental health. BMC Psychiatry 17:1–7 28320380 10.1186/s12888-017-1262-x PMC5359916

[11] Perlman D, Peplau L, Loneliness (1998) Encyclopedia of mental health. New York Academic Press New York

[12] Beutel ME, Klein EM, Brähler E et al (2017) Loneliness in the general population: prevalence, determinants and relations to mental health. BMC Psychiatry 17(1):1–7 28320380 10.1186/s12888-017-1262-x PMC5359916

[13] Cacioppo JT, Hawkley LC, Thisted RA (2010) Perceived social isolation makes me Sad: 5-year cross-lagged analyses of loneliness and depressive symptomatology in the Chicago health, aging, and social relations study. Psychol Aging 25(2):453 20545429 10.1037/a0017216 PMC2922929

[14] Muyan M, Chang EC, Jilani Z, Yu T, Lin J, Hirsch JK (2016) Loneliness and negative affective conditions in adults: is there any room for hope in predicting anxiety and depressive symptoms? J Psychol 150(3):333–341 25970325 10.1080/00223980.2015.1039474

[15] Wang J, Mann F, Lloyd-Evans B, Ma R, Johnson S (2018) Associations between loneliness and perceived social support and outcomes of mental health problems: a systematic review. BMC Psychiatry 18(1):1–16 29843662 10.1186/s12888-018-1736-5 PMC5975705

[16] Holt-Lunstad J, Smith TB, Baker M, Harris T, Stephenson D (2015) Loneliness and social isolation as risk factors for mortality: a meta-analytic review. Perspect Psychol Sci 10(2):227–237 25910392 10.1177/1745691614568352

[17] Christiansen J, Lund R, Qualter P, Andersen CM, Pedersen SS, Lasgaard M (2021) Loneliness, social isolation, and chronic disease outcomes. Ann Behav Med 55(3):203–215 32865550 10.1093/abm/kaaa044

[18] McClelland H, Evans JJ, Nowland R, Ferguson E, O’Connor RC (2020) Loneliness as a predictor of suicidal ideation and behaviour: a systematic review and meta-analysis of prospective studies. J Affect Disord 274:880–896 32664029 10.1016/j.jad.2020.05.004

[19] Kehusmaa J, Ruotsalainen H, Männikkö N et al (2022) The association between the social environment of childhood and adolescence and depression in young adulthood - A prospective cohort study. J Affect Disord 305:37–46. 10.1016/j.jad.2022.02.067 35231482 10.1016/j.jad.2022.02.067

[20] Twenge JM, Haidt J, Blake AB, McAllister C, Lemon H, Le Roy A (2021) Worldwide increases in adolescent loneliness. J Adolesc 93:257–269 34294429 10.1016/j.adolescence.2021.06.006

[21] Parlikar N, Kvaløy K, Strand LB, Espnes GA, Moksnes UK (2023) Loneliness in the Norwegian adolescent population: prevalence trends and relations to mental and self-rated health 10.1186/s12888-023-05404-5 PMC10688064 38037032

[22] Franssen T, Stijnen M, Hamers F, Schneider F (2020) Age differences in demographic, social and health-related factors associated with loneliness across the adult life span (19–65 years): a cross-sectional study in the Netherlands. BMC Public Health 20(1):1–12 32758200 10.1186/s12889-020-09208-0 PMC7409622

[23] Balážová M, Gallová I, Praško J, Šlepecký M, Kotianová A (2017) Family environment as predictor of adolescents’ loneliness. Eur Psychiatry 41(S1):S82–S

[24] Sundqvist A, Hemberg J (2021) Adolescents’ and young adults’ experiences of loneliness and their thoughts about its alleviation. Int J Adolescence Youth 26(1):238–255

[25] Hemberg J, Östman L, Korzhina Y, Groundstroem H, Nyström L, Nyman-Kurkiala P (2022) Loneliness as experienced by adolescents and young adults: an explorative qualitative study. Int J Adolescence Youth 27(1):362–384

[26] Stickley A, Koyanagi A, Koposov R et al (2016) Loneliness and its association with psychological and somatic health problems among Czech, Russian and US adolescents. BMC Psychiatry 16(1):1–11 27146137 10.1186/s12888-016-0829-2 PMC4857285

[27] Jiang J, Wang P (2020) Does early peer relationship last long? The enduring influence of early peer relationship on depression in middle and later life. J Affect Disord 273:86–94 32421625 10.1016/j.jad.2020.04.043

[28] Bruni A, Carbone EA, Pugliese V et al (2018) Childhood adversities are different in schizophrenic spectrum disorders, bipolar disorder and major depressive disorder. BMC Psychiatry 18:1–7 30567512 10.1186/s12888-018-1972-8 PMC6300034

[29] Kehusmaa J, Ruotsalainen H, Männikkö N et al (2022) The association between the social environment of childhood and adolescence and depression in young adulthood-A prospective cohort study. J Affect Disord 305:37–46 35231482 10.1016/j.jad.2022.02.067

[30] Xerxa Y, Rescorla LA, Shanahan L, Tiemeier H, Copeland WE (2023) Childhood loneliness as a specific risk factor for adult psychiatric disorders. Psychol Med 53(1):227–235 34120674 10.1017/S0033291721001422 PMC9874978

[31] Park C, Majeed A, Gill H et al (2020) The effect of loneliness on distinct health outcomes: a comprehensive review and meta-analysis. Psychiatry Res 294:113514 33130511 10.1016/j.psychres.2020.113514

[32] Garmezy N, Masten AS, Tellegen A (1984) The study of stress and competence in children: A Building block for developmental psychopathology. Child Dev.:97–111 6705637

[33] Luthar SS, Cicchetti D, Becker B (2000) The construct of resilience: A critical evaluation and guidelines for future work. Child Dev 71(3):543–562 10953923 10.1111/1467-8624.00164 PMC1885202

[34] Masten AS, Coatsworth JD (1998) The development of competence in favorable and unfavorable environments: lessons from research on successful children. Am Psychol 53(2):205 9491748 10.1037//0003-066x.53.2.205

[35] Werner EE (1995) Resilience in development. Curr Dir Psychol Sci 4(3):81–84

[36] Stainton A, Chisholm K, Kaiser N et al (2019) Resilience as a multimodal dynamic process. Early Interv Psychiat 13(4):725–732 10.1111/eip.12726 30126047

[37] Wingo AP, Wrenn G, Pelletier T, Gutman AR, Bradley B, Ressler KJ (2010) Moderating effects of resilience on depression in individuals with a history of childhood abuse or trauma exposure. J Affect Disord 126(3):411–414 20488545 10.1016/j.jad.2010.04.009 PMC3606050

[38] Ding H, Han J, Zhang M, Wang K, Gong J, Yang S (2017) Moderating and mediating effects of resilience between childhood trauma and depressive symptoms in Chinese children. J Affect Disord 211:130–135. 10.1016/j.jad.2016.12.056 28110160 10.1016/j.jad.2016.12.056

[39] Poole JC, Dobson KS, Pusch D (2017) Childhood adversity and adult depression: the protective role of psychological resilience. Child Abuse Negl 64:89–100. 10.1016/j.chiabu.2016.12.012 28056359 10.1016/j.chiabu.2016.12.012

[40] Hu T, Zhang D, Wang J (2015) A meta-analysis of the trait resilience and mental health. Pers Indiv Differ 76:18–27

[41] Shapero BG, Farabaugh A, Terechina O et al (2019) Understanding the effects of emotional reactivity on depression and suicidal thoughts and behaviors: moderating effects of childhood adversity and resilience. J Affect Disord 245:419–427 30423470 10.1016/j.jad.2018.11.033

[42] Navrady L, Adams M, Chan S, Ritchie S, McIntosh A (2018) Genetic risk of major depressive disorder: the moderating and mediating effects of neuroticism and psychological resilience on clinical and self-reported depression. Psychol Med 48(11):1890–1899 29183409 10.1017/S0033291717003415 PMC6088772

[43] Schulz A, Becker M, Van der Auwera S et al (2014) The impact of childhood trauma on depression: does resilience matter? Population-based results from the study of health in Pomerania. J Psychosom Res 77(2):97–103. 10.1016/j.jpsychores.2014.06.008 25077849 10.1016/j.jpsychores.2014.06.008

[44] Organization WH Adolescent mental health2020

[45] Baciu A, Negussie Y, Geller A, Weinstein JN (2017) National academies of sciences E, medicine. The need to promote health equity. Communities in action: pathways to health equity. National Academies Press (US) 28418632

[46] UNICEF, SUSTAINABLE DEVELOPMENT STARTS AND, ENDS WITH, SAFE (2013) HEALTHY AND WELL-EDUCATED CHILDREN

[47] Holmen TL, Bratberg G, Krokstad S et al (2014) Cohort profile of the Young-HUNT study, Norway: a population-based study of adolescents. Int J Epidemiol 43(2):536–544 23382364 10.1093/ije/dys232

[48] Krokstad S, Langhammer A, Hveem K et al (2013) Cohort profile: the HUNT study, Norway. Int J Epidemiol 42(4):968–977 22879362 10.1093/ije/dys095

[49] Åsvold BO, Langhammer A, Rehn TA et al (2023) Cohort profile update: the HUNT study, Norway. Int J Epidemiol 52(1):e80–e91 35578897 10.1093/ije/dyac095 PMC9908054

[50] Stickley A, Koyanagi A, Koposov R, Schwab-Stone M, Ruchkin V (2014) Loneliness and health risk behaviours among Russian and US adolescents: a cross-sectional study. BMC Public Health 14(1):1–12 24735570 10.1186/1471-2458-14-366 PMC4020347

[51] Peltzer K, Pengpid S (2016) Health risk behaviour among in-school adolescents in the Philippines: trends between 2003, 2007 and 2011, a cross-sectional study. Int J Environ Res Public Health 13(1):73 10.3390/ijerph13010073 PMC4730464 26712770

[52] Rönkä AR, Rautio A, Koiranen M, Sunnari V, Taanila A (2014) Experience of loneliness among adolescent girls and boys: Northern Finland birth cohort 1986 study. J Youth Stud 17(2):183–203

[53] Hjemdal O, Friborg O, Stiles TC, Martinussen M, Rosenvinge JH (2006) A new scale for adolescent resilience: grasping the central protective resources behind healthy development. Meas Evaluation Couns Dev 39(2):84–96

[54] von Soest T, Mossige S, Stefansen K, Hjemdal O (2010) A validation study of the resilience scale for adolescents (READ). J Psychopathol Behav Assess 32:215–225

[55] Kelly Y, Fitzgerald A, Dooley B (2017) Validation of the resilience scale for adolescents (READ) in Ireland: a multi-group analysis. Int J Methods Psychiatr Res 26(2):e1506 27126561 10.1002/mpr.1506 PMC6877176

[56] Moksnes UK, Haugan G (2018) Validation of the resilience scale for adolescents in Norwegian adolescents 13–18 years. Scand J Caring Sci 32(1):430–440 28809052 10.1111/scs.12444

[57] Rosenberg M (2015) Society and the adolescent self-image. Princeton University Press

[58] Schmalbach B, Zenger M, Tibubos AN, Kliem S, Petrowski K, Brähler E (2021) Psychometric properties of two brief versions of the Hopkins symptom checklist: HSCL-5 and HSCL-10. Assessment 28(2):617–631 31272193 10.1177/1073191119860910

[59] Bjerkeset O, Nordahl HM, Larsson S, Dahl AA, Linaker O (2008) A 4-year follow-up study of syndromal and sub-syndromal anxiety and depression symptoms in the general population: the HUNT study. Soc Psychiatry Psychiatr Epidemiol 43:192–199 18064394 10.1007/s00127-007-0289-6

[60] Bjelland I, Dahl AA, Haug TT, Neckelmann D (2002) The validity of the hospital anxiety and depression scale: an updated literature review. J Psychosom Res 52(2):69–77 11832252 10.1016/s0022-3999(01)00296-3

[61] Bjelland I, Dahl AA, Haug TT, Neckelmann D (2002) The validity of the hospital anxiety and depression scale. An updated literature review. J Psychosom Res 52(2):69–77. 10.1016/s0022-3999(01)00296-3 11832252 10.1016/s0022-3999(01)00296-3

[62] Zigmond AS, Snaith RP (1983) The hospital anxiety and depression scale. Acta Psychiatr Scand 67(6):361–370. 10.1111/j.1600-0447.1983.tb09716.x 6880820 10.1111/j.1600-0447.1983.tb09716.x

[63] Redhunt AM, Ledyard R, Ai-ris YC, Hacker MR, Burris HH (2023) Resilience as a potential modifier of Racial inequities in preterm birth. Ann Epidemiol 83:54–59 e1 37088321 10.1016/j.annepidem.2023.04.010 PMC10330189

[64] Collishaw S, Hammerton G, Mahedy L et al (2016) Mental health resilience in the adolescent offspring of parents with depression: a prospective longitudinal study. Lancet Psychiatry 3(1):49–57 26654748 10.1016/S2215-0366(15)00358-2 PMC4703896

[65] Bannink R, Broeren S, van de Looij–Jansen PM, Raat H (2013) Associations between parent-adolescent attachment relationship quality, negative life events and mental health. PLoS ONE 8(11):e80812 24312244 10.1371/journal.pone.0080812 PMC3843678

[66] Andersson T, Alfredsson L, Källberg H, Zdravkovic S, Ahlbom A (2005) Calculating measures of biological interaction. Eur J Epidemiol 20:575–579 16119429 10.1007/s10654-005-7835-x

[67] De Mutsert R, Jager KJ, Zoccali C, Dekker FW (2009) The effect of joint exposures: examining the presence of interaction. Kidney Int 75(7):677–681 19190674 10.1038/ki.2008.645

[68] Rothman KJ (2012) Epidemiology: an introduction. Oxford University Press

[69] VanderWeele TJ, Knol MJ (2014) A tutorial on interaction. Epidemiol Methods 3(1):33–72

[70] Sadock BJ, Sadock VA (2007) Synopsis of psychiatry. Wolters Kluwer

[71] Kessler RC, Sampson NA, Berglund P et al (2015) Anxious and non-anxious major depressive disorder in the world health organization world mental health surveys. Epidemiol Psychiatric Sci 24(3):210–226 10.1017/S2045796015000189 PMC5129607 25720357

[72] Kalin NH (2020) The critical relationship between anxiety and depression. Am Psychiatric Assoc, pp 365–367 10.1176/appi.ajp.2020.20030305 32354270

[73] Cosci F, Fava GA (2021) When anxiety and depression coexist: the role of differential diagnosis using clinimetric criteria. Psychother Psychosom 90(5):308–317 34344013 10.1159/000517518

[74] Maes M, Nelemans SA, Danneel S et al (2019) Loneliness and social anxiety across childhood and adolescence: multilevel meta-analyses of cross-sectional and longitudinal associations. Dev Psychol 55(7):1548 30896228 10.1037/dev0000719

[75] Junker A, Bjørngaard JH, Bjerkeset O (2017) Adolescent health and subsequent risk of self-harm hospitalisation: a 15-year follow-up of the Young-HUNT cohort. Child Adolesc Psychiatry Mental Health 11(1):1–14 10.1186/s13034-017-0161-8 PMC5410696 28469702

[76] Modin B, Östberg V, Almquist Y (2011) Childhood peer status and adult susceptibility to anxiety and depression. A 30-year hospital follow-up. J Abnorm Child Psychol 39(2):187–199. 10.1007/s10802-010-9462-6 20886279 10.1007/s10802-010-9462-6

[77] Lim MH, Eres R, Vasan S (2020) Understanding loneliness in the twenty-first century: an update on correlates, risk factors, and potential solutions. Soc Psychiatry Psychiatr Epidemiol 55:793–810 32524169 10.1007/s00127-020-01889-7

[78] Umberson D, Karas Montez J (2010) Social relationships and health: A flashpoint for health policy. J Health Soc Behav 51(1suppl):S54–S66 20943583 10.1177/0022146510383501 PMC3150158

[79] Mushtaq R, Shoib S, Shah T, Mushtaq S (2014) Relationship between loneliness, psychiatric disorders and physical health? A review on the psychological aspects of loneliness. J Clin Diagn Res 8(9):We01–4. 10.7860/jcdr/2014/10077.4828 25386507 10.7860/JCDR/2014/10077.4828 PMC4225959

[80] Yanguas J, Pinazo-Henandis S, Tarazona-Santabalbina FJ (2018) The complexity of loneliness. Acta Biomed 89(2):302–314. 10.23750/abm.v89i2.7404 29957768 10.23750/abm.v89i2.7404 PMC6179015

[81] Hawkley LC, Hughes ME, Waite LJ, Masi CM, Thisted RA, Cacioppo JT (2008) From social structural factors to perceptions of relationship quality and loneliness: the Chicago health, aging, and social relations study. Journals Gerontol Ser B: Psychol Sci Social Sci 63(6):S375–S84 10.1093/geronb/63.6.s375 PMC2769562 19092047

[82] Hawkley LC, Cole SW, Capitanio JP, Norman GJ, Cacioppo JT (2012) Effects of social isolation on glucocorticoid regulation in social mammals. Horm Behav 62(3):314–323 22663934 10.1016/j.yhbeh.2012.05.011 PMC3449017

[83] Adam EK, Quinn ME, Tavernier R, McQuillan MT, Dahlke KA, Gilbert KE (2017) Diurnal cortisol slopes and mental and physical health outcomes: A systematic review and meta-analysis. Psychoneuroendocrinology 83:25–41 28578301 10.1016/j.psyneuen.2017.05.018 PMC5568897

[84] Doane LD, Adam EK (2010) Loneliness and cortisol: momentary, day-to-day, and trait associations. Psychoneuroendocrinology 35(3):430–441 19744794 10.1016/j.psyneuen.2009.08.005 PMC2841363

[85] Nederhof E, Marceau K, Shirtcliff EA, Hastings PD, Oldehinkel AJ (2015) Autonomic and adrenocortical interactions predict mental health in late adolescence: the TRAILS study. J Abnorm Child Psychol 43:847–861 25421943 10.1007/s10802-014-9958-6

[86] Ruttle PL, Javaras KN, Klein MH, Armstrong JM, Burk LR, Essex MJ (2013) Concurrent and longitudinal associations between diurnal cortisol and body mass index across adolescence. J Adolesc Health 52(6):731–737 23402983 10.1016/j.jadohealth.2012.11.013 PMC3654073

[87] Jopling E, Rnic K, Tracy A, LeMoult J (2021) Impact of loneliness on diurnal cortisol in youth. Psychoneuroendocrinology 132:105345 34229187 10.1016/j.psyneuen.2021.105345

[88] Qualter P, Vanhalst J, Harris R et al (2015) Loneliness across the life span. Perspect Psychol Sci 10(2):250–264 25910393 10.1177/1745691615568999

[89] Shankar A, Hamer M, McMunn A, Steptoe A (2013) Social isolation and loneliness: relationships with cognitive function during 4 years of follow-up in the english longitudinal study of ageing. Psychosom Med 75(2):161–170 23362501 10.1097/PSY.0b013e31827f09cd

[90] Matthews T, Danese A, Caspi A et al (2019) Lonely young adults in modern Britain: findings from an epidemiological cohort study. Psychol Med 49(2):268–277 29684289 10.1017/S0033291718000788 PMC6076992

[91] Matthews T, Danese A, Gregory AM, Caspi A, Moffitt TE, Arseneault L (2017) Sleeping with one eye open: loneliness and sleep quality in young adults. Psychol Med 47(12):2177–2186 28511734 10.1017/S0033291717000629 PMC5551384

[92] Zhang X, Brown AM, Rhubart DC (2023) Can resilience buffer the effects of loneliness on mental distress among working-age adults in the united States during the COVID-19 pandemic? A latent moderated structural modeling analysis. Int J Behav Med 30(6):790–800 36631701 10.1007/s12529-022-10151-0 PMC9838440

[93] Skrove M, Romundstad P, Indredavik MS (2013) Resilience, lifestyle and symptoms of anxiety and depression in adolescence: the Young-HUNT study. Soc Psychiatry Psychiatr Epidemiol 48:407–416 22872359 10.1007/s00127-012-0561-2

[94] Zhao X, Zhang D, Wu M et al (2018) Loneliness and depression symptoms among the elderly in nursing homes: A moderated mediation model of resilience and social support. Psychiatry Res 268:143–151 30025285 10.1016/j.psychres.2018.07.011

[95] Lv F, Yu M, Li J et al (2022) Young adults’ loneliness and depression during the COVID-19 pandemic: A moderated mediation model. Front Psychol 13:842738. 10.3389/fpsyg.2022.842738 35756197 10.3389/fpsyg.2022.842738 PMC9218478

[96] Kalisch R, Müller MB, Tüscher O (2015) A conceptual framework for the Neurobiological study of resilience. Behav Brain Sci 38:e92 25158686 10.1017/S0140525X1400082X

[97] Southwick SM, Charney DS (2012) The science of resilience: implications for the prevention and treatment of depression. Science 338(6103):79–82 23042887 10.1126/science.1222942

[98] Gupta A, Love A, Kilpatrick LA et al (2017) Morphological brain measures of cortico-limbic Inhibition related to resilience. J Neurosci Res 95(9):1760–1775 28029706 10.1002/jnr.24007 PMC5512424

[99] Haddadi P, Besharat MA (2010) Resilience, vulnerability and mental health. Procedia-Social Behav Sci 5:639–642

[100] Marcoen A, Goossens L, Caes P (1987) Lonelines in pre-through late adolescence: exploring the contributions of a multidimensional approach. J Youth Adolesc 16(6):561–577 24277491 10.1007/BF02138821

[101] Mund M, Maes M, Drewke PM, Gutzeit A, Jaki I, Qualter P (2023) Would the real loneliness please stand up? The validity of loneliness scores and the reliability of single-item scores. Assessment 30(4):1226–1248 35246009 10.1177/10731911221077227 PMC10149889

[102] Sauzet O, Ofuya M, Peacock JL (2015) Dichotomisation using a distributional approach when the outcome is skewed. BMC Med Res Methodol 15:1–11 25902850 10.1186/s12874-015-0028-8 PMC4422142

[103] Madsen KR, Holstein BE, Damsgaard MT, Rayce SB, Jespersen LN, Due P (2019) Trends in social inequality in loneliness among adolescents 1991–2014. J Public Health 41(2):e133–e40 10.1093/pubmed/fdy133 30053062

[104] Brennan C, Worrall-Davies A, McMillan D, Gilbody S, House A (2010) The hospital anxiety and depression scale: a diagnostic meta-analysis of case-finding ability. J Psychosom Res 69(4):371–378 20846538 10.1016/j.jpsychores.2010.04.006

[105] Schaffer HR (1996) Social development. Blackwell Publishing

[106] Tambs K, Rønning T, Prescott C et al (2009) The Norwegian Institute of public health twin study of mental health: examining recruitment and attrition bias. Twin Res Hum Genet 12(2):158–168 19335186 10.1375/twin.12.2.158 PMC2743739

[107] Jahre H, Grotle M, Småstuen M et al (2021) Risk factors and risk profiles for neck pain in young adults: prospective analyses from adolescence to young adulthood-The North-Trøndelag health study. PLoS ONE 16(8):e0256006. 10.1371/journal.pone.0256006 34383846 10.1371/journal.pone.0256006 PMC8360564

[108] Wøien Meijer M, Cedergren E, Guðmundsdóttir H (2023) Rooting for the Rural: Changing narratives and creating opportunities for Nordic rural youth

[109] Slätmo E, Norlén G, Dzhavatova K et al (2024) Strategies to Address Nordic Rural Labour Shortage. Nordregio

[110] Langhammer A, Krokstad S, Romundstad P, Heggland J, Holmen J (2012) The HUNT study: participation is associated with survival and depends on socioeconomic status, diseases and symptoms. BMC Med Res Methodol 12:1–14 22978749 10.1186/1471-2288-12-143 PMC3512497

[111] Carpenter JR, Smuk M (2021) Missing data: A statistical framework for practice. Biom J 63(5):915–947. 10.1002/bimj.202000196 33624862 10.1002/bimj.202000196 PMC7615108

