# Supplementary material for: The prospective association of adolescent loneliness and low resilience with anxiety and depression in young adulthood: The HUNT study
Source: Soc Psychiatry Psychiatr Epidemiol. 2025 Apr 7;60(9):2223–35. doi: 10.1007/s00127-025-02888-2 (PMC12378919; doi:10.1007/s00127-025-02888-2)
Supplement: Supplementary file 1 — Supplementary Material 1 [file 127_2025_2888_MOESM1_ESM.docx]

The prospective association of adolescent loneliness and low resilience with anxiety and depression in young adulthood: The HUNT study

**Nayan Parlikar^1*^, Linn Beate Strand^1^, Kirsti Kvaløy^1,2,3^, Geir Arild Espnes^1,^ Unni Karin Mosknes^1^**

Social Psychiatry and Psychiatric Epidemiology

Supplementary data

1. **Sensitivity analyses**
   1. **Multivariable logistic regression**

*Participants scoring ≥ 8 on either question set of HADS (A or D):* We found an association between adolescent loneliness and anxiety and depression in young adulthood **(Table S1)**. Participants with “Often” and “Very often” loneliness at baseline had an OR of 3.46 (95% CI 2.07-5.72) and 4.03 (95% CI 2.13-7.01) respectively for participants scoring ≥ 8 on either question set of HADS (A or D), compared with participants with lesser or without these symptoms after adjusting for age and gender. After adjustment for additional confounders (Model II), the OR was reduced to 2.3 (95% CI 1.34-3.96) and 2.23 (95% CI 1.21-4.12). Additional adjustment for resilience (Model III) resulted in lower OR. Similar associations were noted for *participants scoring ≥ 8 on either question set of HADS (A or D) or with treatment taken for anxiety or depression.* We found an association between adolescents' low resilience and anxiety and depression in young adulthood **(Table S2)**. Participants with low resilience at baseline had an OR of 1.93 (95% CI 1.51-2.47) for participants scoring ≥ 8 on either question set of HADS (A or D) after adjusting for age and gender. After adjustment for confounders (Model II), the OR was reduced to 1.27 (95% CI 0.94-1.7). Additional adjustment for loneliness (Model III) reduced the OR even more. Similar associations were noted for depression and for *participants scoring ≥ 8 on either question set of HADS (A or D) or with treatment taken for anxiety or depression.*

- 1. **Joint associations of adolescent loneliness and low resilience with anxiety and depression in young adulthood**

*Participants scoring ≥ 8 on either question set of HADS (A or D):* In this category, participants with high resilience but experiencing loneliness exhibited an OR of 1.95 (95% CI: 0.92-4.16), whereas those with low resilience but without loneliness exhibited an OR of 1.20 (95% CI: 0.87-1.66). Individuals facing both low resilience and loneliness displayed a notably increased OR of 2.03 (95% CI: 1.23-3.33) **(Table S3)**. The RERI was -0.12 (95% CI -1.82-1.57), showing negative interaction.

*Participants scoring ≥ 8 on either question set of HADS (A or D) or with treatment taken for anxiety or depression:* In this category, participants with high resilience but experiencing loneliness exhibited an OR of 1.67 (95% CI: 0.59-4.68), whereas those with low resilience but without loneliness showed an OR of 1.53 (95% CI: 1.01-2.33). Notably, individuals facing both low resilience and loneliness demonstrated a significantly increased OR of 2.72 (95% CI: 1.33-5.56). There was a positive interaction on the additive scale with a RERI of 0.53 (95% CI -1.94-2.99), for which statistical significance was not observed. The measure of interaction on a multiplicative scale was 1.08 (95% CI 0.29-3.68). This means that there were some indications that the estimated joint effect on the OR scale of loneliness and low resilience together was greater than the product of the estimated effects of loneliness and low resilience alone.

In the next set of sensitivity analyses, after excluding the adolescents with anxiety and depression at baseline (N=1826), 3% (N= 54) reported feeling lonely at baseline, and 13.3% (N= 243) suffered from anxiety and 4.8% (N= 87) endured depression in young adulthood. A higher prevalence of depression was observed among men as compared to women (8.5% men and 7.1% women) **(Tables S4)**. **Table S5-S6** displays the associations of adolescent loneliness and resilience with anxiety and depression in young adulthood performed through multivariable logistic regression analyses. The estimated associations remained fairly unchanged but were less precise, attributable to the limited number of participants within the subcategories of loneliness, anxiety, and depression outcomes. **Table S7** displays the joint associations of adolescent loneliness and low resilience with anxiety and depression. There was positive additive and multiplicative interaction for the outcome measures of depression, for those scoring ≥ 8 on either question set of HADS-A or D and those scoring ≥ 8 on either question set of HADS-A or D or taking treatment for anxiety and depression but did not have statistical significance.

**Table S1:** Loneliness in adolescence in association with anxiety and depression in young adulthood

| **Loneliness** | **Total** | **N (%)** | **Model I**  **OR (95% CI)** | **Model II**  **OR (95% CI)** | **Model III**  **OR (95% CI)** |
| --- | --- | --- | --- | --- | --- |
| **Anxiety or Depression** | | | | | |
| *No* | 901 | 205 (22.8) | Ref | Ref | Ref |
| *A little* | 325 | 122 (37.5) | 2.01 (1.52-2.65) | 1.5 (1.11-2.02) | 1.45 (1.07-1.96) |
| *Quite a lot* | 68 | 34 (50) | 3.46 (2.07-5.72) | 2.3 (1.34-3.96) | 2.09 (1.21-3.63) |
| *Very much* | 56 | 31 (55.4) | 4.03 (2.13-7.01) | 2.23 (1.21-4.12) | 2.03 (1.1-3.77) |
| **Anxiety or Depression or Treatment taken** | | | | | |
| *No* | 450 | 274 (60.9) | Ref | Ref | Ref |
| *A little* | 203 | 158 (77.8) | 2.3 (1.56-3.37) | 1.84 (1.23-2.76) | 1.74 (1.16-2.62) |
| *Quite a lot* | 55 | 45 (81.8) | 2.96 (1.45-6.04) | 2.23 (1.06-4.69) | 1.91 (0.89-4.1) |
| *Very much* | 51 | 44 (86.3) | 4.18 (1.83-9.52) | 3.05 (1.21-7.68) | 2.68 (1.05-6.84) |

Note: CI, confidence interval; HADS-T, Total Hospital Anxiety and Depression scale; OR, odds ratio

Anxiety or Depression: HADS-A scale ≥ 8 or HADS-D ≥ 8

Anxiety or Depression or Treatment taken: HADS-A scale ≥ 8 or HADS-D ≥ 8 or antidepressant medication use or consultation with a psychiatric nurse practitioner or psychiatrist.

Model I adjusted for age, gender

Model II adjusted for age, gender, relationship with family, SES, school satisfaction, and self-esteem at baseline. Model III additionally adjusted for resilience as a continuous variable.

**Table S2:** Resilience measures in adolescence in association with anxiety and depression in young adulthood

| **Resilience** | **Total** | **N (%)** | **Model I**  **OR (95% CI)** | **Model II**  **OR (95% CI)** | **Model III**  **OR (95% CI)** |
| --- | --- | --- | --- | --- | --- |
| **Anxiety or Depression** | | | | | |
| *High* | 992 | 248 (25) | Ref | Ref | Ref |
| *Low* | 409 | 161 (39.4) | 1.93 (1.51-2.47) | 1.27 (0.94-1.7) | 1.13 (0.83-1.53) |
| **Anxiety or Depression or Treatment taken** | | | | | |
| *High* | 503 | 321 (63.8) | Ref | Ref | Ref |
| *Low* | 282 | 223 (79.1) | 2.18 (1.54-3.05) | 1.6 (1.09-2.36) | 1.41 (0.94-2.13) |

Model I adjusted for age, gender

Model II adjusted for age, gender, relationship with family, SES, school satisfaction, and self-esteem at baseline. Model III additionally adjusted for loneliness.

**Table S3:** Adjusted odds ratios for adolescent loneliness and low resilience at baseline associated with anxiety and depression in young adulthood: Additive and multiplicative interaction

| **Loneliness** | **High resilience** | | | **Low Resilience** | | | **RERI (95% CI)** |
| --- | --- | --- | --- | --- | --- | --- | --- |
|  | **Total** | **N** | **OR (95% CI)** | **Total** | **N** | **OR (95% CI)** |  |
| **Anxiety or Depression** | | | | | | | |
| *Rarely lonely* | 921 | 221 | 1.00 | 295 | 105 | 1.2 (0.87-1.66) | -0.12 (-1.82-1.57) |
| *Very lonely* | 32 | 16 | 1.95 (0.92-4.16) | 92 | 49 | 2.03 (1.23-3.33) |  |
| **Anxiety or Depression or Treatment taken** | | | | | | | |
| *Rarely lonely* | 462 | 287 | 1.00 | 188 | 142 | 1.53 (1.01-2.33) | 0.53 (-1.94-2.99) |
| *Very lonely* | 24 | 19 | 1.67 (0.59-4.68) | 82 | 70 | 2.72 (1.33-5.56) |  |

The relative excess risk due to interaction (RERI) between adolescent loneliness (A) and low resilience (B) was calculated using the formula: RERI= ORAB – ORA – ORB + 1.

Adjusted for age, gender, relationship with family, SES, school satisfaction, and self-esteem at baseline

Measure of interaction on the multiplicative scale for anxiety or depression: 0.86 (95% CI. 0.35-2.12)

Measure of interaction on the multiplicative scale for anxiety or depression or treatment taken:1.08 (95% CI 0.29-3.68)

**Table S4:** Baseline (2006-2008) characteristics of the study sample without mental distress (HSCL< 2) stratified on loneliness and symptoms of anxiety and depression in the follow-up (2017-2019)

|  | **Total** | **Young-HUNT3**  **2006-2008** | | **HUNT4**  **2017-2019** | | | |
| --- | --- | --- | --- | --- | --- | --- | --- |
|  |  | Loneliness | | Anxiety symptoms | | Depression symptoms | |
|  |  | No. | % | No. | % | No. | % |
| **Study cohort** | 1826 | 54 | 3 | 243 | 13.3 | 87 | 4.8 |
| **Missing** |  | 104 | 5.7 | 683 | 37.4 | 682 | 37.3 |
| **Gender** |  |  |  |  |  |  |  |
| *Girls* | 972 | 31 | 3.3 | 163 | 23.5 | 49 | 7.1 |
| *Boys* | 854 | 23 | 2.9 | 80 | 17.9 | 38 | 8.5 |
| **Age** |  |  |  |  |  |  |  |
| *13-15 years* | 662 | 19 | 2.9 |  |  |  |  |
| *16-19 years* | 1060 | 35 | 3.3 |  |  |  |  |
| *23-25 years* |  |  |  | 74 | 26.7 | 27 | 9.8 |
| *26-29 years* |  |  |  | 169 | 19.5 | 60 | 6.9 |
| **Resilience** |  |  |  |  |  |  |  |
| *Low* | 364 | 33 | 9.1 | 67 | 27 | 35 | 14.1 |
| *High* | 1349 | 20 | 1.5 | 174 | 19.8 | 52 | 5.9 |
| **Relationship with family** |  |  |  |  |  |  |  |
| *Bad* | 21 | 2 | 9.5 | 5 | 33.3 | 3 | 20 |
| *Good* | 1699 | 52 | 3.1 | 228 | 21.2 | 80 | 7.4 |
| **Self-esteem** |  |  |  |  |  |  |  |
| *Low* | 489 | 37 | 7.6 | 104 | 29.8 | 41 | 11.7 |
| *High* | 1228 | 17 | 1.4 | 138 | 17.5 | 46 | 5.8 |
| **School satisfaction** |  |  |  |  |  |  |  |
| *Not satisfied* | 263 | 22 | 8.4 | 42 | 27.3 | 22 | 14.3 |
| *Satisfied* | 1439 | 29 | 2 | 189 | 20.3 | 57 | 6.1 |
| **Socio-economic status** |  |  |  |  |  |  |  |
| *Low* | 107 | 12 | 11.2 | 19 | 26.8 | 9 | 12.5 |
| *Middle-class* | 265 | 5 | 1.9 | 44 | 25.1 | 14 | 8 |
| *High* | 1335 | 36 | 2.7 | 170 | 20.2 | 58 | 6.9 |

**Table S5:** Loneliness in adolescence in association with anxiety and depression in young adulthood in the study sample without mental distress (HSCL< 2)

| **Loneliness** | **Total** | **N (%)** | **Model I**  **OR (95% CI)** | **Model II**  **OR (95% CI)** | **Model III**  **OR (95% CI)** |
| --- | --- | --- | --- | --- | --- |
| **Anxiety (HADS-A)** | | | | | |
| ***No*** | 834 | 154 (18.5) | Ref | Ref | Ref |
| ***Sometimes*** | 214 | 63 (29.4) | 1.8 (1.27-2.53) | 1.51 (1.05-2.17) | 1.48 (1.03-2.14) |
| ***Often*** | 26 | 26 (42.3) | 3.33 (1.49-7.42) | 2.82 (1.22-6.54) | 2.63 (1.12-6.13) |
| ***Very often*** | 10 | 4 (40) | 2.46 (0.68-8.94) | 2.31 (0.59-9.08) | 2.28 (0.58-8.93) |
| **Depression (HADS-D)** | | | | | |
| ***No*** | 834 | 54 (6.47) | Ref | Ref | Ref |
| ***Sometimes*** | 215 | 21 (9.77) | 1.57 (0.92-2.67) | 1.16 (0.65-2.09) | 1.08 (0.59-1.95) |
| ***Often*** | 26 | 6 (23.1) | 4.53 (1.72-11.93) | 3.31 (1.17-9.38) | 2.68 (0.94-7.62) |
| ***Very often*** | 10 | 2 (20) | 3.07 (0.63-15.12) | 0.92 (0.11-7.92) | 0.89 (0.1-7.78) |
| **Anxiety and Depression (HADS-T)** | | | | | |
| ***No*** | 839 | 38 (4.53) | Ref | Ref | Ref |
| ***Sometimes*** | 217 | 19 (8.76) | 1.99 (1.1-3.5) | 1.51 (1.05-2.17) | 1.46 (0.79-2.8) |
| ***Often*** | 26 | 3 (11.54) | 2.98 (0.85-10.48) | 2.2 (0.57-8.51) | 2.0 (0.52-7.79) |
| ***Very often*** | 10 | 0 (0) | NA | NA | NA |
| **Anxiety or Depression** | | | | | |
| ***No*** | 830 | 174 (21) | Ref | Ref | Ref |
| ***Sometimes*** | 214 | 70 (32.7) | 1.81 (1.29-2.52) | 1.5 (1.05-2.14) | 1.43 (1-2.04) |
| ***Often*** | 26 | 13 (50) | 3.88 (1.76-8.56) | 3.27 (1.42-7.53) | 2.99 (1.25-6.72) |
| ***Very often*** | 10 | 6 (60) | 4.87 (1.34-17.62) | 2.88 (0.74-11.29) | 2.92 (0.74-11.58) |
| **Anxiety or Depression or Treatment taken** | | | | | |
| ***No*** | 400 | 231 (57.76) | Ref | Ref | Ref |
| ***Sometimes*** | 116 | 87 (75) | 2.24 (1.41-3.58) | 1.99 (1.23-3.22) | 1.88 (1.56-3.06) |
| ***Often*** | 18 | 15 (83.33) | 3.7 (1.05-13.03) | 3.26 (0.91-11.65) | 2.57 (0.71-9.35) |
| ***Very often*** | 6 | 6 (100) | NA | NA | NA |

Note: CI, confidence interval; HADS-T, Total Hospital Anxiety and Depression scale; OR, odds ratio

HADS-A scale ≥ 8

HADS-D scale ≥ 8

HADS-A scale ≥ 8 or HADS-D ≥ 8

HADS-T scale ≥ 19

HADS-A scale ≥ 8 or HADS-D ≥ 8 or antidepressant medication use or consultation with a psychiatric nurse practitioner or psychiatrist.

Model I adjusted for age, gender

Model II adjusted for age, gender, relationship with family, SES, school satisfaction, and self-esteem at baseline. Model II additionally adjusted for resilience as a continuous variable.

**Table S6:** Resilience measures in adolescence in association with anxiety and depression in young adulthood in the study sample without mental distress (HSCL< 2)

| **Resilience** | **Total** | | **N (%)** | | **Model I**  **OR (95% CI)** | | **Model II**  **OR (95% CI)** | | **Model III**  **OR (95% CI)** | |
| --- | --- | --- | --- | --- | --- | --- | --- | --- | --- | --- |
| **Anxiety (HADS-A)** | | | | | | | | | | |
| ***High*** | 879 | | 174 (19.8) | | Ref | | Ref | | Ref | |
| ***Low*** | 248 | | 67 (27) | | 1.49 (1.07-2.07) | | 1.32 (0.91-1.91) | | 1.22 (0.78-1.47) | |
| **Depression (HADS-D)** | | | | | | | | | | |
| ***High*** | 880 | | 52 (5.9) | | Ref | | Ref | | Ref | |
| ***Low*** | 248 | | 35 (14.1) | | 2.63 (1.66-4.13) | | 2.21 (1.29-3.78) | | 2.08 (1.2-3.58) | |
| **Anxiety and Depression (HADS-T)** | | | | | | | | | | |
| ***High*** | 885 | | 40 (4.5) | | Ref | | Ref | | Ref | |
| ***Low*** | 252 | | 22 (8.7) | | 1.99 (1.16-3.44) | | 1.48 (0.78-2.82) | | 1.41 (0.74-2.69) | |
| **Anxiety or Depression** | | | | | | | | | | |
| ***High*** | 875 | | 192 (22) | | Ref | | Ref | | Ref | |
| ***Low*** | 247 | | 81 (32.8) | | 1.73 (1.26-2.36) | | 1.53 (1.07-2.17) | | 1.41 (0.98-2.03) | |
| **Anxiety or Depression or Treatment taken** | | | | | | | | | | |
| ***High*** | | 416 | | 248 (59.6) | | Ref | | Ref | | Ref |
| ***Low*** | | 139 | | 104 (74.8) | | 2.03 (1.32-3.13) | | 1.78 (1.13-2.85) | | 1.68 (1.04-2.73) |

Note: CI, confidence interval; HADS-T, Total Hospital Anxiety and Depression scale; OR, odds ratio

HADS-A scale ≥ 8

HADS-D scale ≥ 8

HADS-T scale ≥ 19

Anxiety or Depression: HADS-A scale ≥ 8 or HADS-D ≥ 8

Anxiety or Depression or Treatment taken: HADS-A scale ≥ 8 or HADS-D ≥ 8 or antidepressant medication use or consultation with a psychiatric nurse practitioner or psychiatrist.

Model I adjusted for age, gender

Model II adjusted for age, gender, relationship with family, SES, school satisfaction, and self-esteem at baseline. Model III additionally adjusted for loneliness.

**Table S7:** Adjusted odds ratios for adolescent loneliness and low resilience at baseline associated with anxiety and depression in young adulthood in the study sample without mental distress (HSCL< 2) : Additive and multiplicative interaction

| **Loneliness** | **High resilience** | | | **Low Resilience** | | | **RERI (95% CI)¹** |
| --- | --- | --- | --- | --- | --- | --- | --- |
|  | **Anxiety symptoms (HADS-A)** | | | | | |  |
|  | **Total** | **N** | **OR (95% CI)** | **Total** | **N** | **OR (95% CI)** |  |
| *Rarely lonely* | 830 | 160 | 1.00 | 213 | 57 | 1.28 (0.86-1.88) | -0.18 (-3.58-3.23) |
| *Very lonely* | 15 | 6 | 2.37 (0.78-7.17) | 21 | 9 | 2.46 (0.97-6.27) |  |
| **Depression symptoms (HADS-D)** | | | | | | | |
| *Rarely lonely* | 831 | 48 | 1.00 | 213 | 27 | 1.95 (1.1-3.44) | 3.65 (-2.25-9.54) |
| *Very lonely* | 15 | 2 | 0.85 (0.1-7.07) | 21 | 6 | 5.45 (1.84-16.10) |  |
| **Anxiety or Depression** | | | | | | | |
| *Rarely lonely* | 827 | 177 | 1.00 | 212 | 67 | 1.42 (0.98-2.06) | 1.66 (-2.67-5.98) |
| *Very lonely* | 15 | 7 | 2.0 (0.66-6.07) | 21 | 12 | 4.07 (1.59-10.47) |  |
| **Anxiety and Depression (HADS T)** | | | | | | | |
| *Rarely lonely* | 835 | 38 | 1.00 | 216 | 19 | 1.47 (0.75-2.86) | -0.09 (-3.54-3.37) |
| *Very lonely* | 15 | 1 | 1.17 (0.14-10.1) | 21 | 2 | 1.55 (0.31-7.71) |  |
| **Anxiety or Depression or Treatment taken** | | | | | | | |
| *Rarely lonely* | 396 | 231 | 1.00 | 119 | 86 | 1.67 (1.03-2.69) | 7.14 (-12.22-26.49) |
| *Very lonely* | 9 | 7 | 1.59 (0.30-8.39) | 15 | 14 | 9.39 (1.21-72.94) |  |

¹The relative excess risk due to interaction (RERI) between adolescent loneliness (A) and low resilience (B) was calculated using the formula: RERI= ORAB – ORA – ORB + 1.

Adjusted for age, gender, relationship with family, SES, school satisfaction, and self-esteem at baseline

Measure of interaction on the multiplicative scale (HADS-A): 0.78 (95% CI. 0.18-3.43)

Measure of interaction on the multiplicative scale (HADS-D): 3.29 (95% CI 0.41-71.65)

Measure of interaction on the multiplicative scale (HADS-T): 0.9 (95% CI 0.07-22.71)

Measure of interaction on the multiplicative scale for anxiety or depression: 1.43 (95% CI. 0.32-6.24)

Measure of interaction on the multiplicative scale for anxiety or depression or treatment taken: 3.49 (95% CI 0.26-88.87)
